# Berberine Alleviates Kainic Acid‐Induced Acute Epileptic Seizures in Mice via Reshaping Gut Microbiota‐Associated Lipid Metabolism

**DOI:** 10.1111/cns.70253

**Published:** 2025-02-06

**Authors:** Wen‐Ting Dai, Yong Zhu, Zui‐Ming Jiang, Yi Xiang, Xiao‐Yuan Mao, Zhao‐Qian Liu

**Affiliations:** ^1^ Department of Clinical Pharmacology, Hunan Key Laboratory of Pharmacogenetics, and National Clinical Research Center for Geriatric Disorders Xiangya Hospital, Central South University Changsha China; ^2^ Institute of Clinical Pharmacology, Engineering Research Center for Applied Technology of Pharmacogenomics of Ministry of Education Central South University Changsha China; ^3^ Department of Clinical Laboratory, The Affiliated Zhuzhou Hospital Xiangya Medical College Central South University Zhuzhou Hunan China; ^4^ Blood Transfusion Department, The Affiliated Zhuzhou Hospital Xiangya Medical College Central South University Zhuzhou Hunan China

**Keywords:** acute epilepsy, Berberine, gut microbiota, lipidomics

## Abstract

**Background:**

Berberine (BBR) has been reported to mitigate epileptic seizures. However, the potential mechanism of its anti‐seizure effect remains uncharacterized.

**Aims:**

This study aimed to investigate the protective effect of BBR on acute epileptic seizures induced by kainic acid (KA) in mice and further explore its mechanism of action in the aspect of analysis of gut microbiota.

**Materials and Methods:**

The protective effect of BBR against acute epileptic seizures was assessed via Racine score and Nissl training. Alterations of gut microbiota and metabolites in seizure mice after BBR treatment were analyzed through 16S sequencing and lipidomics, respectively.

**Results:**

Our results showed that the BBR remarkably alleviated acute epileptic seizures and hippocampal neuron damage in KA‐induced mice. The analysis of gut microbiota indicated that BBR reduced the acute epileptic seizures in KA‐induced mice by increasing the abundance of Bacteroidetes and Alloprevotella, regulating short‐chain fatty acids (SCFAs). Results of lipidomics also identified 21 candidate metabolites in the colon and hippocampus possibly involved in the protective effect of BBR against acute seizures.

**Conclusion:**

These findings suggest that BBR exerts neuroprotection against KA‐induced epileptic seizures through remodeling gut microbiota‐associated lipid metabolism in the colon and hippocampus. BBR may serve as a valuable candidate drug for curing patients with epilepsy.

## Graphical abstract

BBR exerts promising protection against KA‐induced epileptic seizures through remodeling gut microbiota to regulate lipid metabolism in the colon and hippocampus.

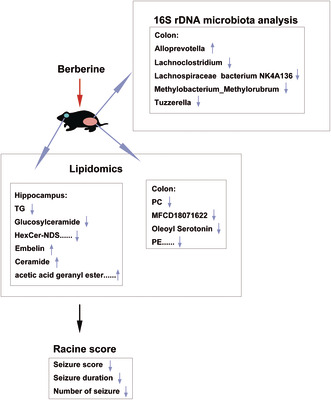

## 1 Introduction

To date, more than 70 million people suffer from epilepsy around the world. Overall, this neurologic condition occurs in both male and female groups except that localization‐related symptomatic epilepsies and cryptogenic localization‐related epilepsies are more prevalent in men and women, respectively [1]. It is well established that epilepsy remains a neurological disorder characterized by the appearance of spontaneous recurrent seizures and associated cognitive, psychological, and social comorbidities [2]. Epilepsy is characterized by excessive, hypersynchronous neuronal firing, primarily manifested by interactions between cortical and subcortical structures [3]. Glutamate is the main excitatory neurotransmitter in the mammalian central nervous system [4]. Excessive accumulation of Glu may cause neuronal hyperexcitability by binding to ionotropic Glu (IGLU) receptors and involve in epileptogenesis [5]. On the one hand, glutamate is converted into GABA by glutamate decarboxylase (GAD). Decreased GABA level and/or elevated Glu content results in disruption of the balance of inhibitory and excitatory neurotransmission, finally facilitating the etiology of epilepsy [6]. On the other hand, glutamate is taken up by astrocytes and converted to glutamine by glutamine synthetase (GS). Defects or dysfunction of astrocytic GS diminishes glutamate clearance and triggers several types of epilepsy, including drug‐resistant mesial temporal lobe epilepsy [7], neocortical epilepsy [8], and glioma‐related epilepsy [9].

The pathological origins of epilepsy are still largely unknown but seem to include neural network reorganization, neurogenesis, neuroinflammation, excitoxicity, axon germination, and cell death [10, 11]. Neuroinflammation is a well‐recognized contributing factor in the pathophysiology of epilepsy. It has been demonstrated that neuroinflammatory signals trigger epileptic seizures and this phenomenon also leads to the activation of inflammation‐related cells including astrocytes and microglia and the release of inflammatory factors, forming a vicious circle [12]. The dysregulation of gut microbiota composition and destruction of blood–brain barrier (BBB) permeability can exacerbate this vicious cycle. The gut microbiota may mediate the peripheral inflammation through immune system activation (e.g., the release of inflammatory cytokines and chemokines), thus participating in the occurrence of epilepsy. Usually, gut microbiota has the capacity to produce diverse neurotransmitters (e.g., serotonin [5‐HT] and γ‐aminobutyric acid [GABA]) and other metabolites, including short‐chain fatty acids (SCFAs) to regulate the neural network, thereby affecting the excitation and inhibition (E/I) balance [13, 14].

There are several strategies for counteracting epileptic seizures in a clinic. Anti‐seizure drugs (ASDs) are the major medications for epilepsy. However, approximately 30% of patients with epilepsy are refractory to the conventional ASDs [15]. Although more than 40 ASMs have been approved since the 1980s, the number of drug‐resistant epilepsy patients is still increasing [16]. Surgical resection may be a useful therapeutic avenue for some epilepsy patients, but it is applicable for a limited number of patients and is usually invasive. In addition, ketogenic diet (KD) and vagus nerve stimulator implantation have also shown therapeutic implications [17], but it is limited to some population groups. Therefore, there is an urgent need to develop innovative anti‐epileptic strategies to improve the progression and/or abrogate the harmful consequences of the disease [2, 18].

The gut microbiota is a complex symbiotic microbiota, which is colonized in the gastrointestinal tract [19]. Under physiological conditions, the gut microbiota plays a crucial role in regulating intestinal permeability, changing local/peripheral immune response, and producing essential metabolites and neurotransmitters [18, 20]. In most healthy individuals, 99% of the gut microbiota is composed of *Firmicutes*, *Bacteroidota*, *Proteobacteria*, and *Actinobacteria*, with *Firmicutes* and *Bacteroidota* accounting for approximately 90% of the total microbiota [21, 22, 23]. Lee et al. found that the number of *Bacteroidetes* in the epilepsy group was lower than that in the healthy group and an increase of *Actinobacteria* was observed in the healthy group. Compared with the healthy group, the microbial community richness index of the epileptic subjects was 1.6–1.7 times lower, and they had a unique species composition [24].

The metabolic products of gut microbiota are the key molecular mediators between gut microbiota and host [25]. It has been demonstrated that gut microbiota can affect the glutamine–glutamate–GABA cycle in various ways, namely by producing neurotransmitters and amino acids and regulating the expressions of GABA and NMDA receptors in some brain regions such as the hippocampus, amygdala, and locus coeruleus [26].

Berberine (BBR) is an isoquinoline alkaloid isolated from Rhizoma Coptidis and other Berberis plants, which has a wide clinical application for intestinal diseases [27]. Recently, it has also been reported to alleviate epileptic seizures in rodent models [28]. In addition, BBR and its derivatives can restore the expression of c‐fos, a biomarker for neuronal excitation, and neuronal discharge during PTZ‐induced epileptic seizures [28]. These studies suggest that BBR has therapeutic potential against epilepsy. However, the concrete anti‐seizure mechanism of it is not well understood.

In the present study, the protective effect of BBR on kainic acid (KA)‐induced acute seizures in mice was evaluated and whether gut microbiota was involved in its anti‐seizure potential was further investigated.

## 2 Materials and Methods

### 2.1 Animals

Male C57BL/6J mice (6–8 weeks) were bred in the Laboratory Animal Center of Central South University. All mice were housed under a standard environment (temperature at the range of 20°C–24°C, 12 h light/12 h dark cycle) with free access to food and water. Experimental procedures were approved by Animal Care and Use Committee of Xiangya Hospital of Central South University (Approval number: 2022020555). A total of 165 male C57BL/6J mice were used in this study, and 30 mice died.

### 2.2 Drug Administration

BBR (HY‐18258) was obtained from MedChem Express (MCE, New Jersey, USA). BBR was dissolved in ddH_2_O containing 5% DMSO. Mice were administrated BBR (25 mg/kg body weight) or an equal volume of vehicle by intragastric administration once a day for 14 consecutive days. Injection of KA was carried out after the last administration of BBR. KA (1 μL, 250 ng/μL dissolved in saline) provided by MedChem Express (HY‐N2309, MCE, New Jersey, USA) was stereotaxically injected into the hippocampus on the basis of the following coordinates: anteroposterior −2.0 mm; lateral −1.3 mm; dorsoventral −1.2 mm.

### 2.3 Antibiotics Treatment

C57BL/6 mice were intragastrically treated with vancomycin (50 mg/kg, V2002, Sigma), neomycin (100 mg/kg, HY‐B0470, MedChem Express), and metronidazole (100 mg/kg, HY‐B0318, MedChem Express) solutions twice a day for 7 days. Ampicillin (1 mg/mL, A5354, Sigma) was added freely in the drinking water. In the control group, mice were intragastrically treated with drinking water twice a day for 7 days. Antibiotics‐treated mice were fed sterile food and water in sterile cages.

### 2.4 Preparation of KA‐Induced Acute Epileptic Seizures

Intrahippocampal injection of KA into mice was performed for induction of epileptic seizures. Following anesthesia with isoflurane, mice were fixed onto the stereotactic instrument. The scalp was carefully cut along the midline of the head and the anterior chimney was exposed. According to mouse brain atlas, the location of the hippocampus was precisely determined (anteroposterior −2.0 mm; lateral −1.3 mm; dorsoventral −1.2 mm). KA (250 ng/μL) was slowly injected for 10 min along the drilling hole. After that, the puncture needle was removed and the skull was sealed with bone wax. Seizure severity was assessed using Racine scoring system as elaborated in the following section.

### 2.5 Racine Score

Seizure behavior was assessed by the Racine Scale according to previous study [29]. The detailed criteria were shown as follows: stage 0, no reaction; stage 1, rhythmic twitching of the face and beard; stage 2, head bobbing and circling; stage 3, multiple limb myoclonus and spasm; stage 4, rising and falling; stage 5, generalized tonic–clonic seizures accompanied by running and jumping; and stage 6, death. Mice with the third or higher seizure stage were considered to be successful establishment of the epileptic seizure model and selected in subsequent experiments.

### 2.6 Transplantation of Fecal Microbiota

Fresh fecal samples were collected from donor mice fed with BBR or NC for 14 days and suspended in ice‐cold phosphate‐buffered saline (PBS) at 50 mg/mL. Antibiotics‐treated mice were colonized by oral gavage of 100 μL suspension. For mock treatment, mice were gavaged with PBS. The mice were placed in miniature isolation cages and treated aseptically. Seizure score was assessed 4 days after transplantation.

### 2.7 Nissl Staining

To explore the effect of BBR on seizure‐induced neuronal damage, mice were randomly divided into six groups as follows: (1) Control group (*n* = 6): After the intragastric administration with physiological saline 2 h, 1 μL PBS was injected stereotaxically, and then intragastric administration with physiological saline once a day for 3 days; (2) KA group (*n* = 6): After the intragastric administration with physiological saline 2 h, 1 μL KA (250 ng/μL) was injected stereotaxically, and then subject to intragastric administration of physiological saline once a day for 3 days; (3) KA + BBR group (*n* = 6): After the intragastric administration with BBR (25 mg/kg body weight) 2 h, 1 μL KA (250 ng/μL) was injected stereotaxically, and then intragastric administration of BBR once a day for 3 days; (4) Abx + KA + BBR group (*n* = 6): After treatment with the antibiotics treatment for 7 days and the intragastric administration with BBR (25 mg/kg body weight) 2 h, 1 μL KA (250 ng/μL) was injected stereotaxically, and then intragastric administration of BBR once a day for 3 days. Brain tissue sections (10 μm thickness) from each group were incubated with Nissl staining solution (C0117, Beyotime Biotechnology, Shanghai, China) for 10 min. After dehydration, the brain slides were covered with neutral balm and captured under an optical microscope (Leica, Wetzlar, Germany). Nissl bodies in CA1 and CA3 subregions of hippocampus were calculated using Image J software (Bethesda, Maryland, USA), as these two regions are susceptible to neuronal damage after KA‐induced acute epileptic seizures.

### 2.8 16S rDNA Microbiota Analysis

The fecal samples of C57BL/6 mice administered with BBR or vehicle were collected. DNA was immediately isolated from 250 mg of feces, and the remaining samples were aliquots and stored at −80°C. Fecal samples of mice were treated with aseptic technology under a BSL2 laminar flow hood. Microbial DNA was isolated from fecal samples using QIAamp PowerFecal DNA Kit and QIAmp DNA Stool Mini Kit (Qiagen, Germantown, MD), respectively. DNA concentration was measured using Nanodrop‐ND1000, and samples were stored at −80°C in the refrigerator. The preparation and sequencing of 16S rRNA gene amplification library were carried out in Nuohezhuyuan Laboratory (Beijing, China). The V4 region of the 16S rRNA gene (515F‐806R) was amplified by PCR using region‐specific primers containing sequence adaptor sequences for Illumina Flowcell. The forward amplification primer also contains a 12‐base bar code sequence, supporting the pooling of up to 2167 different samples in each channel. The amplicons were quantified using PicoGreen (Invitrogen) and tablet reader (Infinite 200 PRO, Tecan) and sequenced on Illumina MiSeq. The amplification sequence of 16S rRNA gene was analyzed by BLAST search of NCBI bacterial nucleotide sequence database. Alpha diversity and beta diversity were analyzed with QIIME package and R software (version 2.15.3). Alpha diversity was used to analyze the complexity of sample species diversity through 3 metrics, including observed species, Shannon diversity index, and Simpson diversity index. Beta diversity analysis was used to assess the differences in species complexity among swab samples. The GUniFrac package in R language (v4.1.3) was used for weighted Unifrac distance matrix and principal coordinate analysis (PCoA). The difference between the two samples was analyzed by T‐test.

### 2.9 Quantitative Polymerase Chain Reaction (qPCR) for Microbiota Genera

Bacterial genomic DNA was extracted from mouse fecal samples or colonic lumen contents using a mobio powerOil kit, where sample size reflected the independent cages of 3 mice in each cage. The V4 region of 16S rDNA gene was amplified by PCR with universal primers and 30 ng of genomic DNA. The PCR reaction was carried out in triplicate, and the PCR products were purified with a Qiaquick PCR Purification Kit (Qiagen). The purified PCR products were quantified by Kapa library quantitative Kit (Kapa Biosystems, KK4824), and paired‐end sequencing was performed using Illumina Miseq platform and kit. The genera abundance of *Alloprevotella*, *Lachnoclostridium*, *Lachnospiraceae NK4A136*, *Tuzzerella*, and *Methylobacterium_Methylorubrum* were detected in the NC and BBR groups by qPCR. Primer sequences to detect these genera were designed and listed in Table 1. Primers were designed based on the sequence encoding 16S rRNA from the NCBI‐BLAST for specific detection of *Alloprevotella* (ACCESSION AJ005634), *Lachnoclostridium* (ACCESSION CAWUZH010000003.1), *Lachnospiraceae bacterium NK4A136* (Accession: ATVW00000000.1), *Tuzzerella* (ACCESSION NZ_CAUWHO010000117.1), and *Methylobacterium_Methylorubrum* (Accession: NZ_BAAADH010000107.1).

**TABLE 1:** PCR primer sequences used in the present study.

Bacteria	Primer sequence
Alloprevotella	Forward: 5′‐CGAGTTGTCCAGCGAAAGCG‐3′
Alloprevotella	Reverse: 5′‐CTGCACTCCGCACTCTTGGT‐3′
Lachnoclostridium	Forward: 5′‐CGACGTGCAGAGAGACATGG‐3′
Lachnoclostridium	Reverse: 5′‐CGGCGATGATCTCGTCGATC‐3′
Lachnospiraceae NK4A136	Forward: 5′‐CCACATTGGGACTGAGACAC‐3′
Lachnospiraceae NK4A136	Reverse: 5′‐CTTTCGAGCCTCAACGTCAG‐3′
Tuzzerella	Forward: 5′‐CTCTGTTGTCTATTTTAAATTGTTT‐3′
Tuzzerella	Reverse: 5′‐TATAATAAAATTTTTGAGGCAGAC‐3′
Methylobacterium_Methylorubrum	Forward: 5′‐GGCTTAACACATGCAAGTCGA‐3′
Methylobacterium_Methylorubrum	Reverse: 5′‐TTGCGGTTAGCGCAGCG‐3′

### 2.10 Colonic Lumenal and Hippocampal Lipidomics

The colonic contents were collected from the mouse terminal colon, immediately frozen in liquid nitrogen, and stored at −80°C. The hippocampal tissue samples were homogenized in 1 mL of cold 80% methanol and vigorously mixed on ice before centrifugation (4359 g, 4°C). The 5 μg supernatant was transferred to a glass bottom and dissolved in 100 μL isopropyl alcohol. The samples were analyzed using the Vanquish UHPLC system (Thermo Fisher, Germany) and Orbitrap Q ActivateTM HF mass spectrometer (Thermo Fisher, Germany). The Compound Discoverer 3.01 (CD3.1, Thermo Fisher) was used to process the original data files generated by UHPLC–MS/MS for peak comparison, peak selection, and quantification for each metabolite. Peaks were matched with Lipidmaps and Lipidblast databases to obtain accurate qualitative and relatively quantitative results. Statistical software R (R version R‐3.4.3), Python (Python version 2.7.6), and CentOS (CentOS version 6.6) were used for statistical analysis.

### 2.11 Statistical Analysis

Except for the specific statistical analysis used in the lipidomics and microbiology experiments, data were represented as mean ± SEM. One‐way ANOVA or repeated measure (RM)‐two‐way ANOVA with Tukey's test and Sidak's test were used to determine the statistical significance of normally distributed data. With respect to the data not normally distributed, Mann–Whitney *U* test or Kruska–Wallis test was employed. *p* < 0.05 was considered to possess a significant difference. The detailed information on the statistical test is summarized in Table 2.

**TABLE 2:** Summary of statistical tests.

Figure	Statistical test
1A	Two‐way ANOVA, *F*(1, 42) = 209.3, Alpha = 0.05
1B	Two‐way ANOVA, *F*(1, 42) = 1105, Alpha = 0.05
1C	Two‐way ANOVA, *F*(1, 42) = 821, Alpha = 0.05
1D	Kruskal–Wallis test, statistic = 16.91, *p* value = 0.0002
1E	Two‐way ANOVA, *F*(2, 12) = 229.0, Alpha = 0.05
1F	Two‐way ANOVA, *F*(2, 14) = 359.2, Alpha = 0.05
2A	Kruskal–Wallis test, statistic = 17.01, *p* value = 0.0002
2B	Two‐way ANOVA, *F*(2, 14) = 228.8, Alpha = 0.05
2C	Two‐way ANOVA, *F*(2, 14) = 229.9, Alpha = 0.05
2D	Two‐way ANOVA, *F*(3, 16) = 176.5, Alpha = 0.05
5A	Unpaired *t* test, two‐tailed: *t* = 3.304, *df* = 4, Alpha = 0.05
5B	Unpaired *t* test, two‐tailed: *t* = 11.86, *df* = 4, Alpha = 0.05
5C	Unpaired *t* test, two‐tailed: *t* = 8.143, *df* = 4, Alpha = 0.05
5D	Unpaired *t* test, two‐tailed: *t* = 3.453, *df* = 4, Alpha = 0.05
5F	Unpaired *t* test, two‐tailed: *t* = 5.162, *df* = 4, Alpha = 0.05
6C	One‐way ANOVA followed by LSD post hoc test, FC = 0.23878025, *p* value = 3.09024E‐05
6D	One‐way ANOVA followed by LSD post hoc test, FC = 0.426527419, *p* value = 8.34461E‐06
6E	One‐way ANOVA followed by LSD post hoc test, FC = 0.323422805, *p* value = 9.251E‐06
6F	One‐way ANOVA followed by LSD post hoc test, FC = 0.151713138, *p* value = 7.65214E‐08
6G	One‐way ANOVA followed by LSD post hoc test, FC = 0.230092615, *p* value = 1.10614E‐05
6H	One‐way ANOVA followed by LSD post hoc test, FC = 0.056149668, *p* value = 5.79401E‐05
6I	One‐way ANOVA followed by LSD post hoc test, FC = 0.087883724, *p* value = 1.30776E‐05
6J	One‐way ANOVA followed by LSD post hoc test, FC = 0.109240075, *p* value = 1.19075E‐05
8C	One‐way ANOVA followed by LSD post hoc test, FC = 1.724502447, *p* value = 0.000214664
8D	One‐way ANOVA followed by LSD post hoc test, FC = 2.683459929, *p* value = 0.000145787
8E	One‐way ANOVA followed by LSD post hoc test, FC = 1.927417311, *p* value = 6.57027E‐06
8F	One‐way ANOVA followed by LSD post hoc test, FC = 15.70299913, *p* value = 4.12217E‐05
8G	One‐way ANOVA followed by LSD post hoc test, FC = 1.957957647, *p* value = 0.000153892
8H	One‐way ANOVA followed by LSD post hoc test, FC = 1.764929897, *p* value = 0.000291369
8I	One‐way ANOVA followed by LSD post hoc test, FC = 0.599145175, *p* value = 0.000110992
8J	One‐way ANOVA followed by LSD post hoc test, FC = 0.654081464, *p* value = 0.000225999
8K	One‐way ANOVA followed by LSD post hoc test, FC = 2.570201875, *p* value = 0.000236828
8L	One‐way ANOVA followed by LSD post hoc test, FC = 0.585079192, *p* value = 4.01142E‐05
8M	One‐way ANOVA followed by LSD post hoc test, FC = 0.423548755, *p* value = 2.24196E‐05
8N	One‐way ANOVA followed by LSD post hoc test, FC = 0.581214862, *p* value = 7.03585E‐05
8O	One‐way ANOVA followed by LSD post hoc test, FC = 2.022871284, *p* value = 6.67065E‐05
8P	One‐way ANOVA followed by LSD post hoc test, FC = 3.200786952, *p* value = 0.000182335
8Q	One‐way ANOVA followed by LSD post hoc test, FC = 9.036504519, *p* value = 0.00029402
8R	One‐way ANOVA followed by LSD post hoc test, FC = 1.96127266, *p* value = 8.06454E‐05
8S	One‐way ANOVA followed by LSD post hoc test, FC = 10.2473592, *p* value = 7.86415E‐05

## 3 Results

### 3.1 Gut Microbiota Is Involved in the Anti‐Seizure Effect of BBR

**FIGURE 1:**
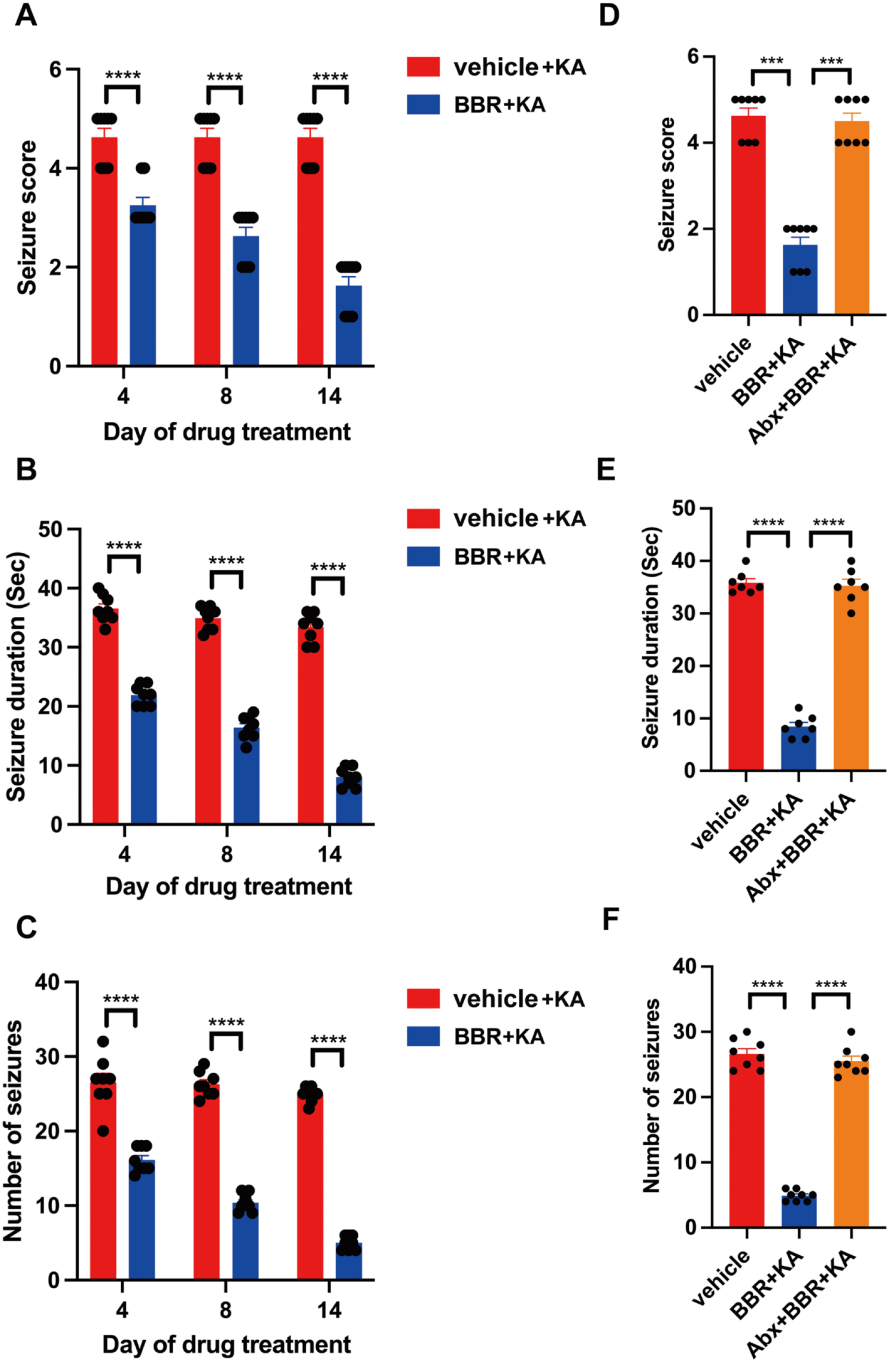
Deletion of microbiota blocks the antiseizure effect of BBR. (A) Seizure stage after 4, 8, and 14 day of BBR administration, *n* = 8 per group (NC), 8 per group (BBR); (B) seizure duration after 4, 8, and 14 day of BBR administration, *n* = 8 per group (NC), 8 per group (BBR); (C) number of seizures after 4, 8, and 14 day of BBR administration, *n* = 8 per group (NC), 8 per group (BBR); (D) seizure stage after 7 days of antibiotic treatment and 14 days after BBR administration, *n* = 8 per group; (E) seizure duration after 7 days of antibiotic treatment and 14 days after BBR administration, *n* = 8 per group; (F) the number of seizures after 7 days of antibiotic treatment and 14 days after BBR administration, *n* = 8 per group. All the data were expressed as mean ± SEM, ****p < 0.0001.

After BBR (25 mg/kg) was orally treated for 4, 8, and 14 days, KA was subject to intrahippocampal injection of KA once and seizure phenotype was assessed. It was found that BBR significantly reduced seizure score (Figure 1A), seizure duration (Figure 1B), and number of seizures (Figure 1C) at different time points. However, treatment with antibiotics evidently abrogated antiseizure potential of BBR including seizure score (Figure 1D), seizure duration (Figure 1E), and the number of seizures (Figure 1F). These data suggest that the gut microbiota mediates the antiseizure effect of BBR.

### 3.2 The Gut Microbiota Provides Seizure Protection to Mice Treated With BBR‐Associated Gut Microbes

**FIGURE 2:**
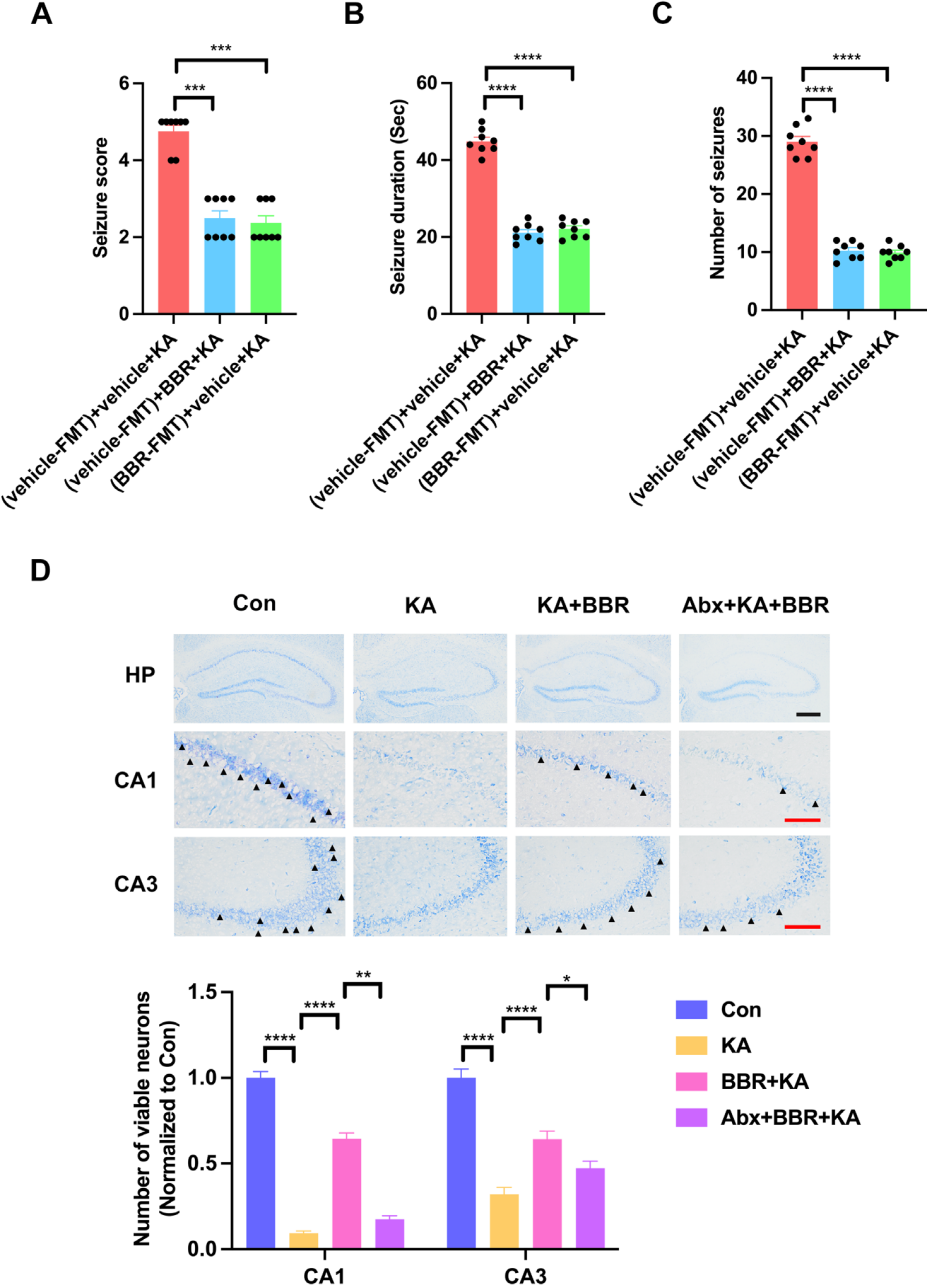
The bacteria associated with BBR fully exerted seizure protection in mice fed with NC. (A) Seizure stage in response to KA in ABX‐treated SPF mice transplanted with NC microbiota (NC‐FMT) or BBR microbiota (BBR‐FMT) and treated with NC or BBR, *n* = 8 per group; (B) seizure duration in response to KA in ABX‐treated SPF mice transplanted with NC microbiota (NC‐FMT) or BBR microbiota (BBR‐FMT) and treated with NC or BBR, *n* = 8 per group; (C) the number of seizures in response to KA in ABX‐treated SPF mice transplanted with NC microbiota (NC‐FMT) or BBR microbiota (BBR‐FMT) and treated with NC or BBR, *n* = 8 per group; (D) representative images of Nissl staining and statistical analysis of viable neurons in the hippocampus demonstrated the effects of BBR and gut microbiota on the survival neurons of KA‐treated mice, *n* = 5 per group. The black scale bar represents 200 μm; The red scale bar represents 50 μm. All the data were presented as mean ± SEM, *p < 0.05, **p < 0.01, ***p < 0.001, ****p < 0.0001.

To determine whether BBR‐associated gut microbes also confer antiseizure effects, Abx‐treated mice were transplanted with NC versus BBR microbiota from SPF mice and tested their susceptibility to KA seizures after 4 days of NC or BBR treatment. Compared to transplanting NC microbiota and treatment with NC, transplanting NC microbiota and treatment with BBR could significantly reduce seizure score (Figure 2A), seizure duration (Figure 2B), and number of seizures (Figure 2C), which was consistent with the findings showing the protective effect of BBR against epileptic seizures. Importantly, compared to the control group transplanted with NC microbiota, the transplantation of BBR microbiota significantly reduced seizure score (Figure 2A), seizure duration (Figure 2B), and number of seizures (Figure 2C) in mice treated with NC. It indicates that the BBR‐associated microbiota provides seizure protection in mice treated with NC. We further investigated the effect of BBR on seizure‐induced neuronal damage by Nissl staining analysis. The results of Nissl staining showed that KA significantly reduced the survival neurons in CA1 and CA3 subregions of hippocampus, and BBR significantly reversed this phenomenon (Figure 2D). These results indicate that treatment with BBR‐associated microbes exhibits protection against epileptic seizures.

### 3.3 Changes of Gut Microbial Composition and Taxa in Mice Administered With BBR

**FIGURE 3:**
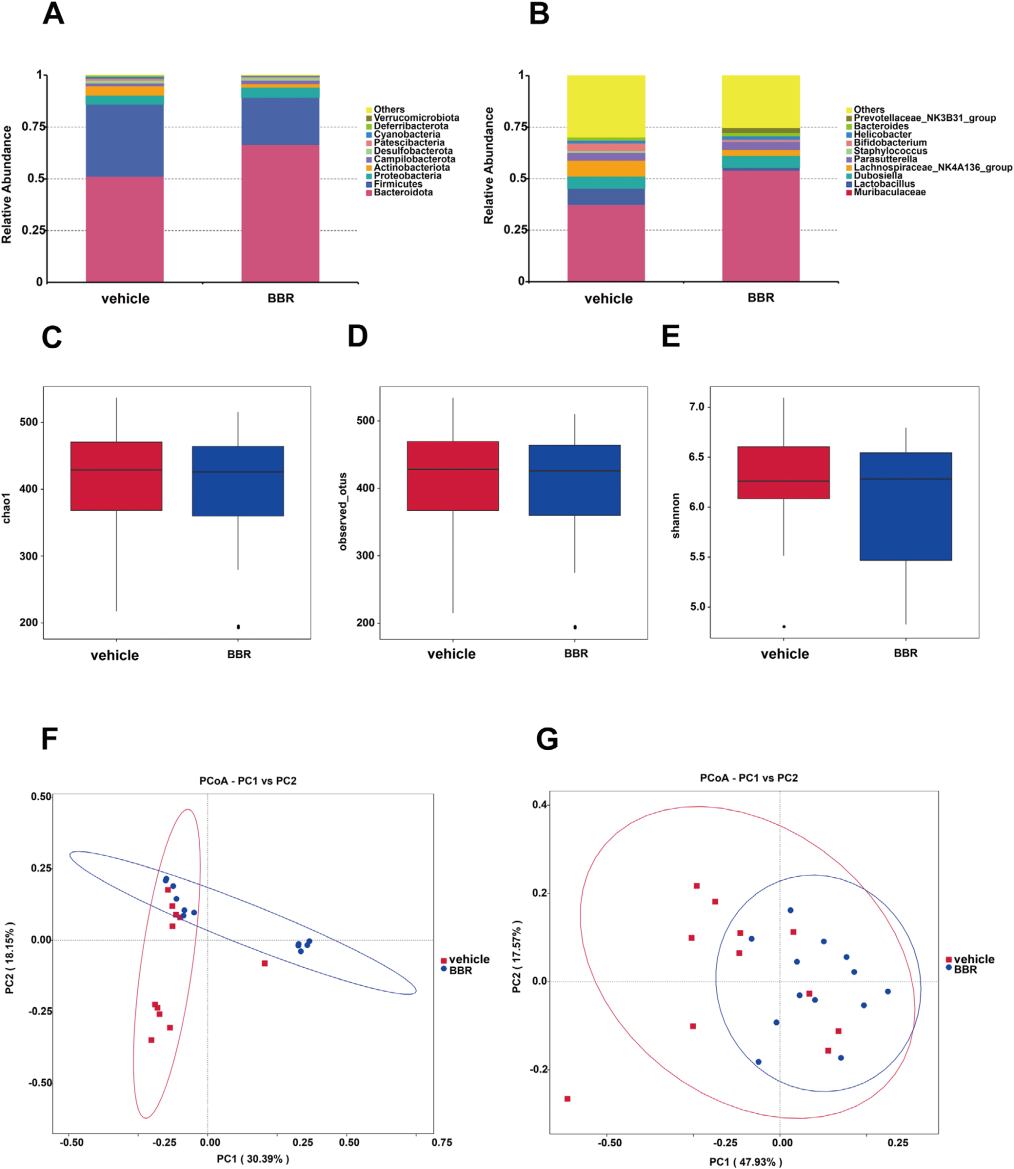
Gut microbial biodiversity analysis in BBR‐ and NC‐treated mice. (A and B) Abundance (% V3–V4 of 16S rRNA sequence) of the top 10 dominant phylum and genus at 4, 8, and 14 days after administration of NC and BBR. Each block diagram represented the median, interquartile spacing, minimum, and maximum values. There was no statistical difference; (C–E) the box diagram showed the alpha‐diversity measure within three indicators (chao1, observed OTU number, and shannon index). No statistical difference was found; (F and G) beta diversity was observed by the principal coordinate analysis of unweighted and weighted Unifrac dissimilarity matrices. The first and second principal coordinates of these two measures were reported. One‐way ANOVA or Kruskal–Wallis test was used to determine the *p*‐value.

**FIGURE 4:**
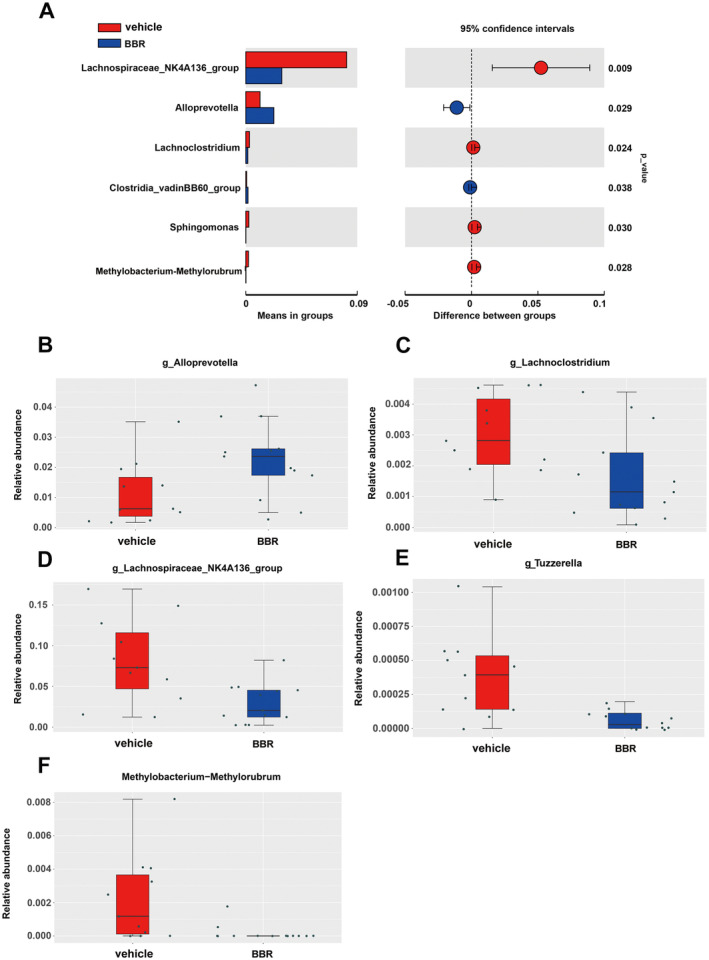
Microbial community dynamics in mice treated with BBR for 14 days. (A) Statistical analysis of microbiota in BBR and NC groups; (B) the relative abundance of Alloprevotella was increased in the BBR group; (C–F) in the BBR treatment group, the relative abundance of Lachnoclostridium, Lachnospiraceae_NK4A136_group, Tuzzerella, and Methylobacterium_Methylorubrum were decreased (paired Wilcoxon rank sum test).

**FIGURE 5:**
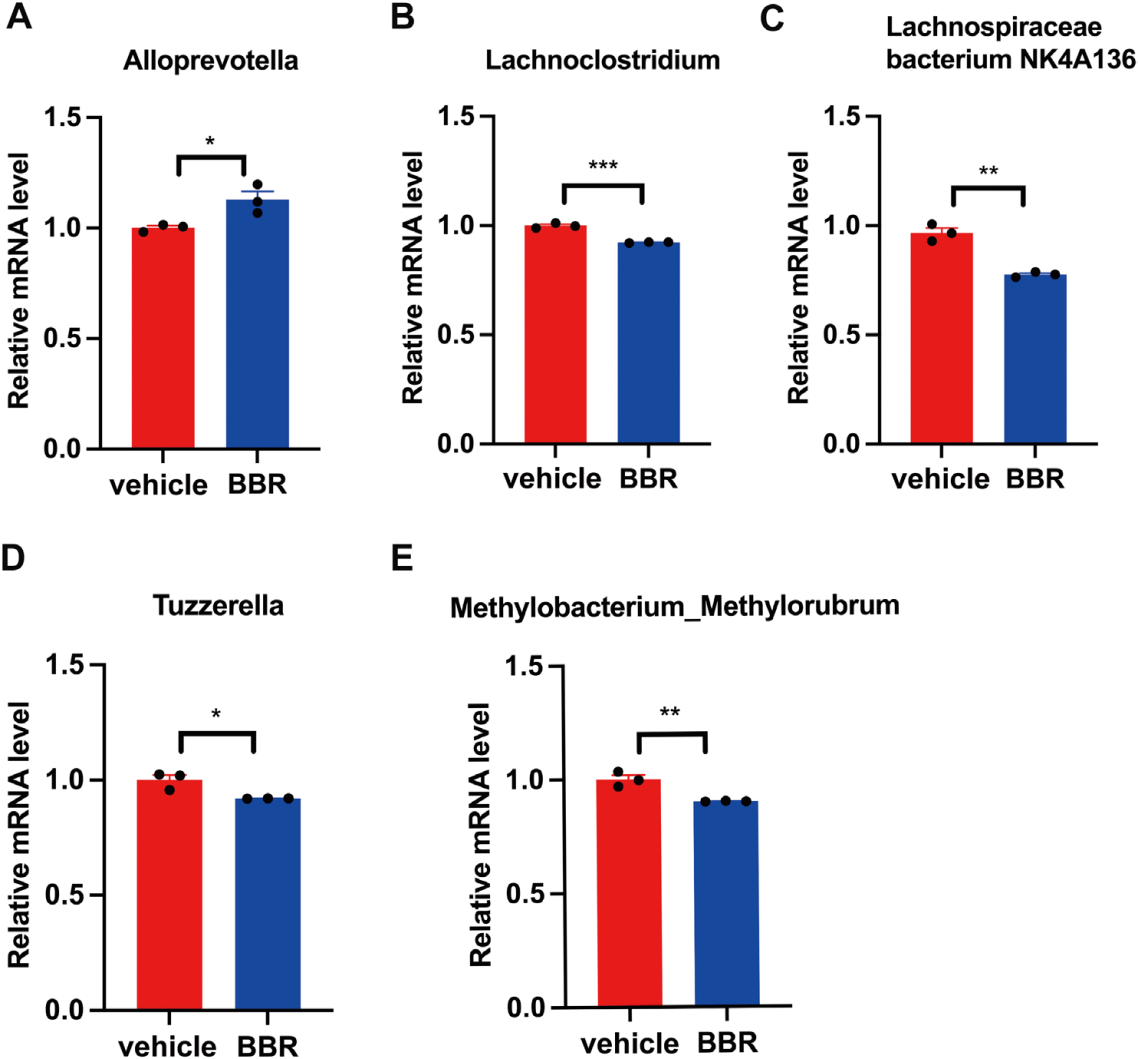
qPCR was used to verify the differential gut microbiota in the NC and BBR groups. (A–E) indicated the abundance of Alloprevotella, Lachnoclostridium, Lachnospiraceae_NK4A136_group, Tuzzerella, and Methylobacterium_ Methylorubrum, respectively, in NC and BBR groups. Data were displayed as mean ± SEM, *p < 0.05, **p < 0.01, ***p < 0.001.

Through 16S rRNA gene sequencing, gut microbiota was screened in BBR‐ or vehicle‐treated mice. It was found that the *Bacteroidota* was increased at the phylum level, while the amount of *Firmicutes* and *Actinobacteriota* was decreased after BBR treatment. Meanwhile, the *Prevotellaceae_NK3B31_grou*p was increased at the genus level (Figure 3A,B), while the amount of *Lachnospiraceae_NK4A136_group*, *Bifidobacterium*, and *Lactobacillus* was diminished. In addition, the α diversity of gut microbiota was decreased after BBR treatment at different time points (Figure 3C–E). The results of β diversity analysis revealed that the gut microbiota of SPF mice treated with BBR was similar to that of SPF mice treated with NC (Figure 3F,G). The relative abundance of related taxa between the BBR‐ and NC‐treated groups was also compared in our present work. It was found that after 14 days of BBR treatment, the abundance of *Alloprevotella* was elevated at the genus level (Figure 4A,B), but the abundance of *Lachnoclostridium*, *Lachnospiraceae_NK4A136_group*, *Tuzzerella*, and *Methylobacterium_Methylorubrum* was decreased at the genus level (Figure 4C–F). To validate these significantly different genus results obtained from 16S rRNA sequence data, qPCR on five genera was further carried out. In the most dominant genera, there was an increase in the abundance of *Alloprevotella* at the genus level (Figure 5A), and a decrease in the abundance of *Lachnoclostridium*, *Lachnospiraceae_NK4A136_group*, *Tuzzerella*, and *Methylobacterium_Methylorubrum* at the genus level (Figure 5B–E). Taken together, these data suggest that BBR treatment results in the alteration of gut microbiota.

### 3.4 Colonic Lumenal Lipidomics Analysis

**FIGURE 6:**
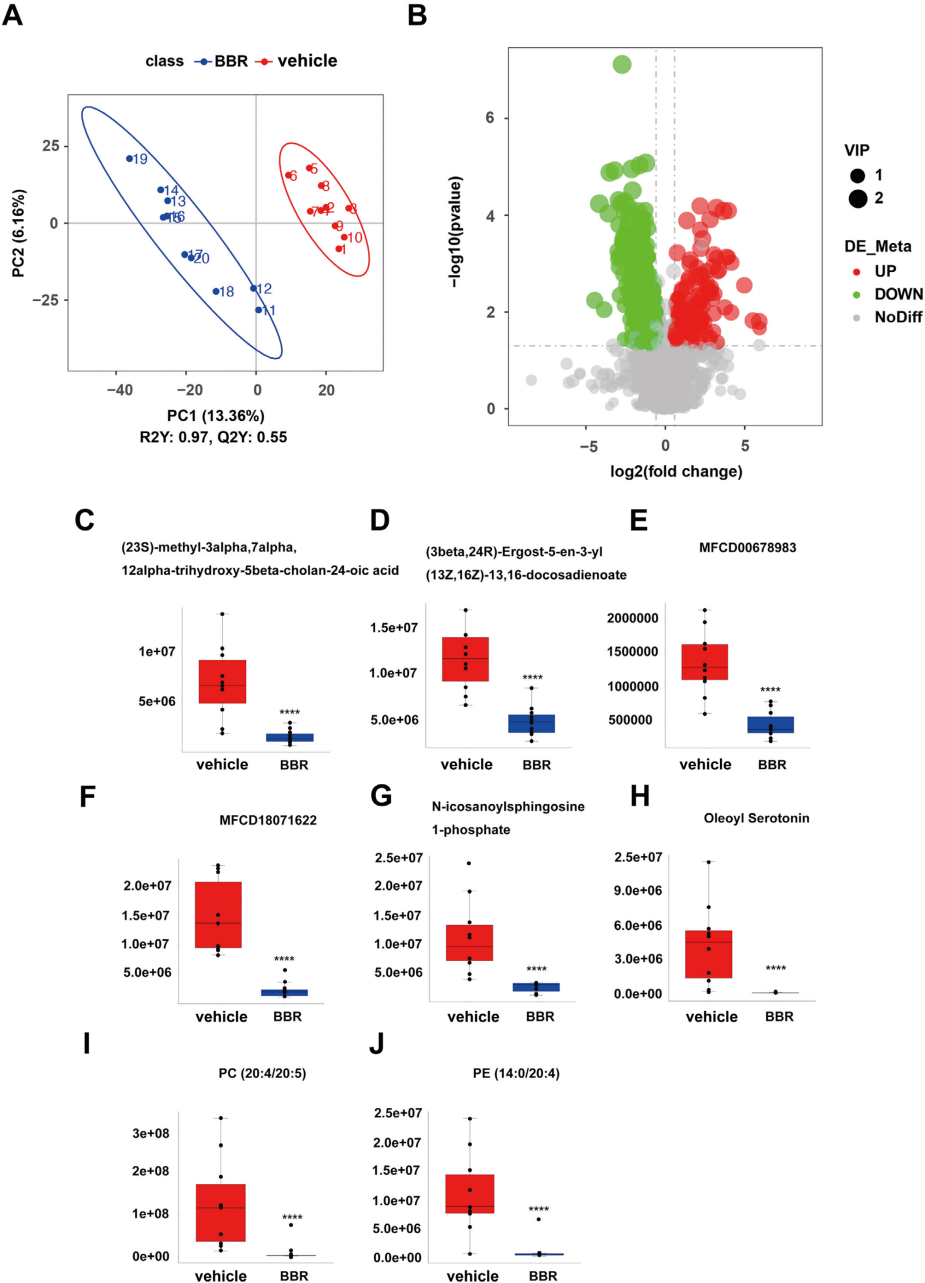
Analysis of lipid profiles in mice colon treated with BBR and NC by lipidomics. (A) PLS‐DA score maps for two groups, with the horizontal axis representing the score of the sample on the first principal component. The vertical axis represented the score of the sample on the second principal component. R2Y represented the explanatory power of the model. Q2Y was utilized to assess the predictive ability of the PLS‐DA model, and when R2Y was greater than Q2Y, it indicated that the model was well established. (B) Volcano plots of two groups, with the horizontal axis representing the fold change of lipid compounds in different groups (log_2_(fold change)) and the vertical axis representing the level of significant difference (−log_10_(*p*‐value)). Each point in the plot represented a lipid compound, and the size of the points represented the VIP value. Significant upregulated and downregulated lipid metabolites were indicated by red and green dots, respectively; (C–J) Changes of lipid metabolism in two groups. Statistical significance was assessed by one‐way ANOVA followed by an LSD post hoc test. ****p < 0.0001.

**FIGURE 7:**
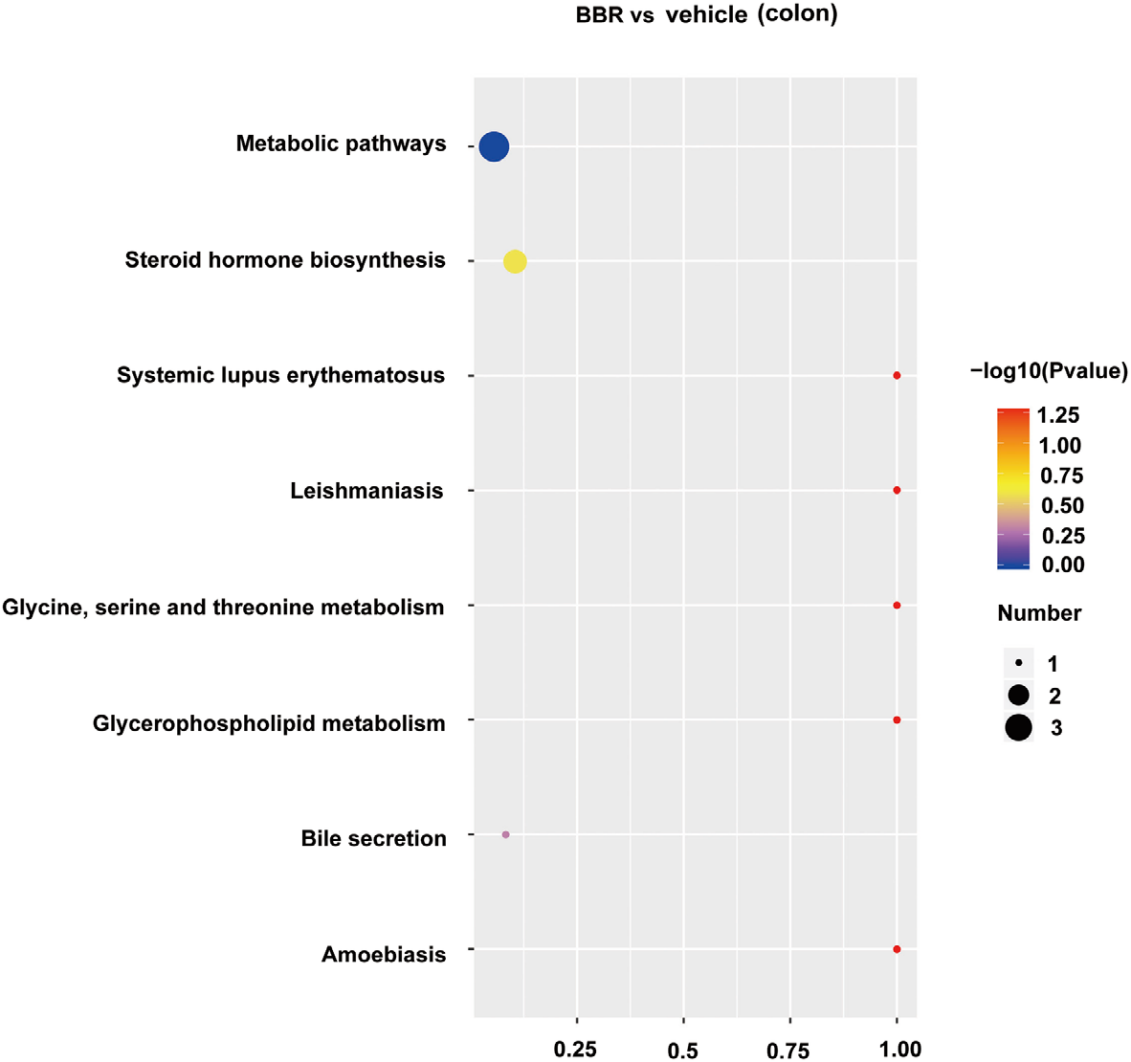
KEGG analysis of lipid profiles in mice colon treated with BBR and NC. With the help of a hypergeometric test, the *p*‐value value of the pathway enrichment was obtained, with a threshold of *p*‐value at 0.05. KEGG pathway satisfying this condition was defined as KEGG pathway significantly enriched in different lipid compounds. The size of the dot represents the number of differential lipid compounds in the corresponding pathway. The larger the dot, the more differential lipid compounds in the pathway.

PLS‐DA analysis was used to determine changes in lipid distribution in the colon of the BBR group. The validation parameters of two multivariate PLS‐DA models include the fitness (R2Y = 0.97) and predictability (Q2Y = 0.55) of NC and BBR, indicating that the model has good fitness and can be considered as a predictable model (Figure 6A). Based on the criteria of FC > 1.5 or < 0.667, VIP > 1, and *p* < 0.05, candidate lipid biomarkers were screened using volcano plot analysis. The results showed that 338 significantly differential lipids were identified in NC and BBR, with 101 upregulation and 237 downregulation (Figure 6B). The changed levels and nomenclature of colonic lipid metabolites related to differential gut microbiota are shown in Figure 6. Compared with the NC group, (23S)‐methyl‐3alpha,7alpha,12alpha‐trihydroxy‐5beta‐cholan‐24‐oic acid, (3beta,24R) ‐Ergost‐5‐en‐3‐yl (13Z,16Z)‐13,16‐docosadienoate, MFCD00678983, MFCD 18071622, N‐icosanoylsphingosine 1‐phosphate, oleoyl serotonin, PC (20:4/20:5), and PE (14:0/20:4) were significantly decreased in the BBR group. KEGG pathway analysis was employed to enrich the pathways of the screened lipids, as shown in Figure 7. The top 8 metabolic pathways are displayed in Figure 7, based on *p*‐value (from small to large). Glycine, serine, and threonine metabolism and glycerophospholipid metabolism were the potential pathways.

### 3.5 Hippocampal Lipidomics Analysis

**FIGURE 8:**
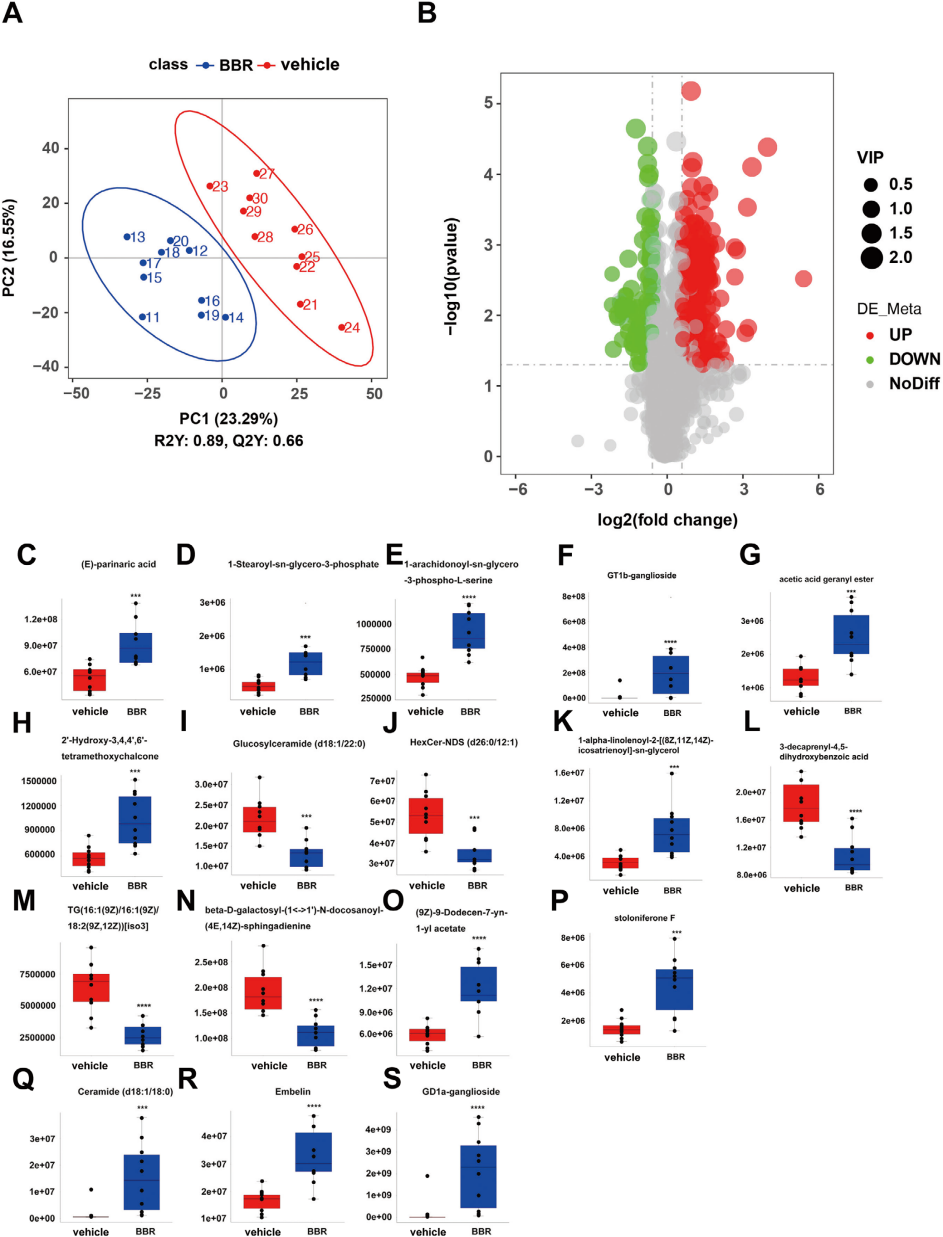
Analysis of lipid profiles in mice hippocampus treated with BBR and NC by lipidomics. (A) Indication of PLS‐DA with the horizontal axis representing the score of the sample on the first principal component and the score of the sample on the second principal component by the vertical axis. R2Y represented the explanatory power of the model and Q2Y was used to evaluate the predictive ability of the PLS‐DA model, and when R2Y was greater than Q2Y, it indicated that the model was well established; (B) Volcano plots of two groups, with the horizontal axis representing the fold change of lipid compounds in different groups (log_2_(fold change)) and the vertical axis representing the level of significant difference (−log_10_(*p*‐value)). Each point in the plot represented a lipid metabolite, and the size of the points represented the VIP value. Significantly upregulated and downregulated lipids were marked by red and green dots, respectively; (C–T) Alterations of lipid metabolism in two groups. Statistical significance was assessed by one‐way ANOVA followed by an LSD post hoc test. ***p < 0.001, ****p < 0.0001.

**FIGURE 9:**
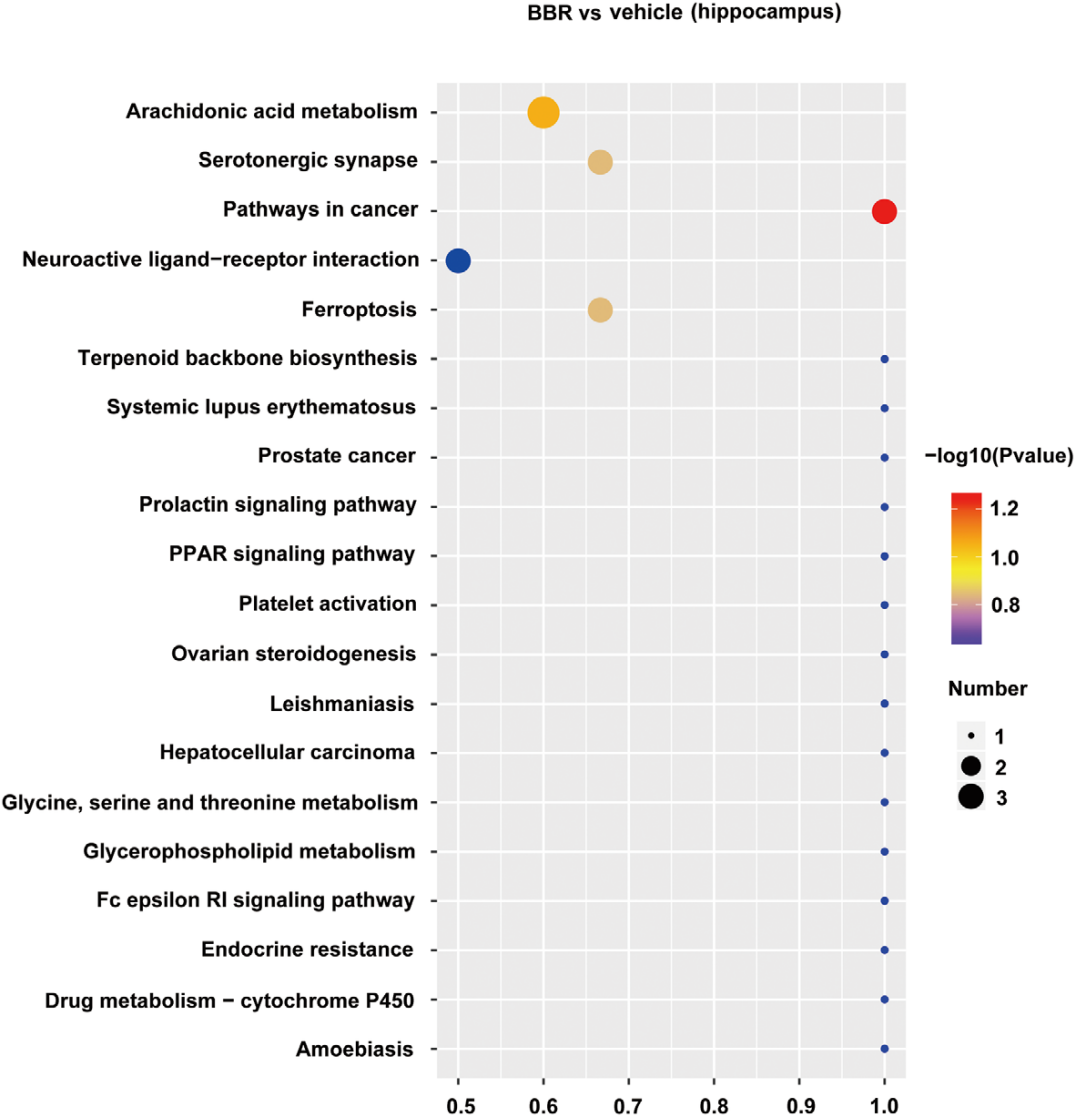
KEGG analysis of lipid profiles in mice hippocampus treated with BBR and NC. Following the enrichment analysis, there are multiple lipid‐associated pathways identified. The top 20 pathways are indicated with the significance set at *p* value less than 0.05.

There were 160 identified significantly differential lipids in NC and BBR‐treated groups, with 131 upregulation and 29 downregulation (Figure 8). The changed levels and nomenclature of hippocampal lipid metabolites related to differential gut microbiota are shown in Figure 8. Compared with the NC group, (E)‐parinaric acid, 1‐stearoyl‐sn‐glycero‐3‐phosphate, 1‐alpha‐linolenoyl‐2‐[(8Z,11Z,14Z)‐icosatrienoyl]‐sn‐glycerol, 2′‐hydroxy‐3,4,4′,6′‐tetramethoxychalcone, (9Z)‐9‐dodecen‐7‐yn‐1‐yl acetate, ceramide (d18:1/18:0), embelin, GD1a‐ganglioside, GT1b‐ganglioside, acetic acid geranyl ester, 1‐alpha‐linolenoyl‐2‐[(8Z,11Z,14Z)‐icosatrienoyl]‐sn‐glycerol, and stoloniferone F were significantly increased in the BBR group. Nevertheless, glucosylceramide (d18:1/22:0), HexCer‐NDS (d26:0/12:1), TG (16:1(9Z)/16:1(9Z)/18:2(9Z,12Z))[iso3], beta‐D‐galactosyl‐(1↔1′)‐N‐ docosanoyl‐(4E,14Z)‐sphingadienine, and 3‐decaprenyl‐4,5‐dihydroxybenzoic acid were significantly decreased in the BBR group. The pathways of the screened lipids in hippocampal tissue samples were obtained via KEGG analysis, as shown in Figure 9. The top 20 metabolic pathways are displayed in Figure 9, based on *p*‐value. Arachidonic acid metabolism, serotonergic synapse, and ferroptosis were the potential pathways.

### 3.6 Colonic Lumenal Lipids Were Altered After BBR Treatment and Associated With the Gut Microbiota

**FIGURE 10:**
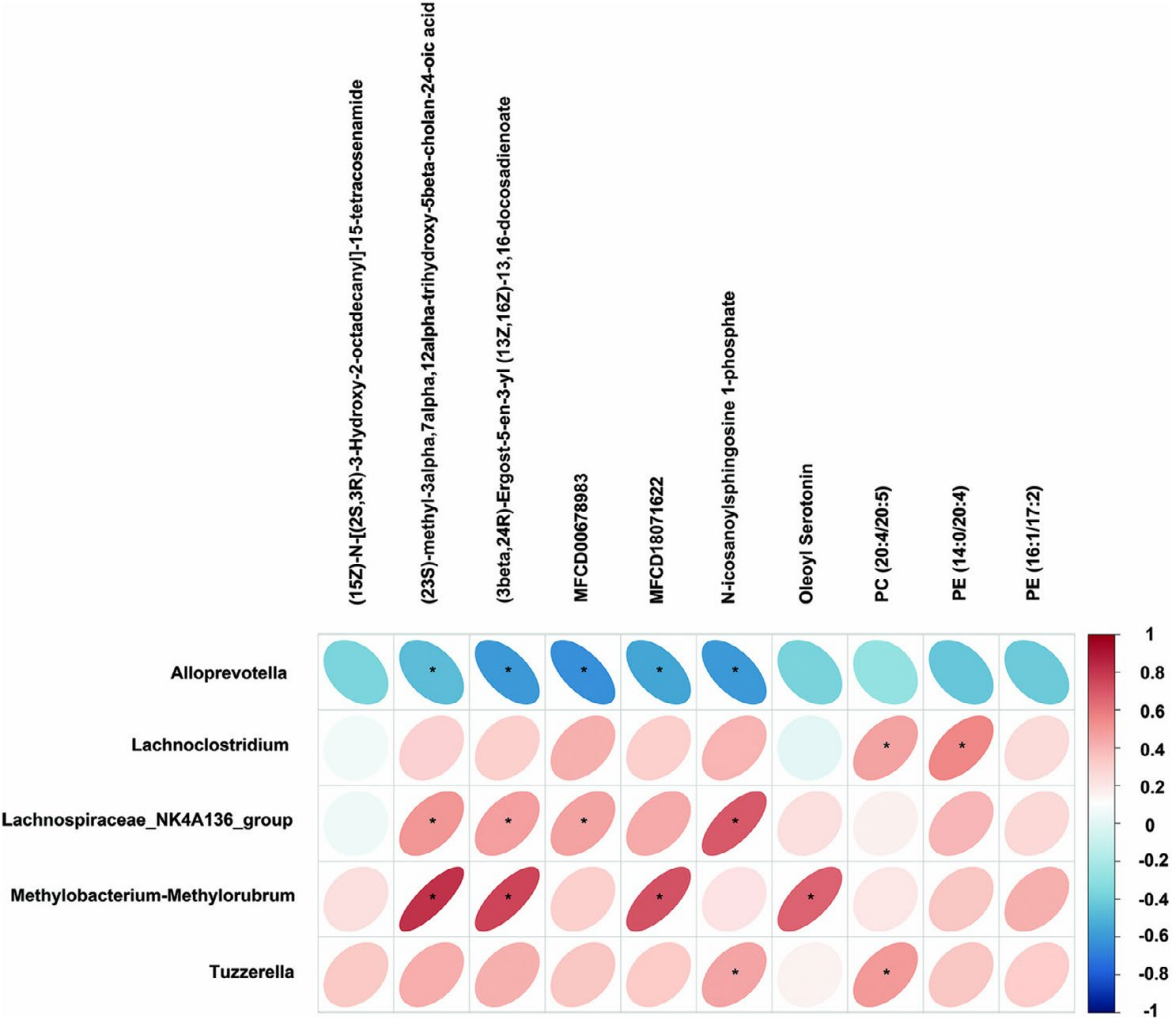
The correlation analysis between gut microbiota and lipid metabolites in colon. The heatmap showed the correlation coefficient between gut microbiota at the genus level and colonic lipid metabolite levels. Horizontal represented differential bacterial genera and vertical represented differential metabolites. The red represented the positive correlation, while the blue represented the negative correlation. Pearson statistical method, *p < 0.05.

Pearson correlation was utilized to explore the association with diverse taxonomic groups at the genus level and the Colonic lumenal lipids (Figure 10). *Lachnospiraceae_NK4A136_group* and *Methylobacterium‐Methylorubrum* were positively correlated with (23S)‐methyl‐3alpha,7alpha,12alpha ‐trihydroxy‐5beta‐cholan‐24‐oic acid, (3beta,24R)‐Ergost‐5‐en‐3‐yl (13Z,16Z)‐13, 16‐docosadienoate, while *Alloprevotella* was negatively correlated with (23S)‐methyl‐3alpha,7alpha,12alpha‐trihydroxy‐5beta‐cholan‐24‐oic acid, (3beta,24R)‐Ergost‐5‐en‐3‐yl (13Z,16Z)‐13,16‐docosadienoate. *Lachnospiraceae_NK4A136_group* had a positive correlation with MFCD00678983, while *Alloprevotella* had a negative correlation. Moreover, *Methylobacterium‐Methylorubrum* was positively correlated with MFCD18071622, while *Alloprevotella* had a negative correlation. *Lachnospiraceae_NK4A136_group* and *Tuzzerella* were positively correlated with N‐icosanoylsphingosine 1‐phosphate, while *Alloprevotella* was negatively correlated with it. In addition, we also observed a strong correlation between *Methylobacterium‐Methylorubrum* and Oleoyl Serotonin. *Lachnoclostridium* was highly correlated with PE (14:0/20:4). *Tuzzerella* and *Lachnoclostridium* were positively correlated with PC (20:4/20:5).

### 3.7 Hippocampal Lipids Were Altered After BBR Treatment and Associated With the Gut Microbiota

**FIGURE 11:**
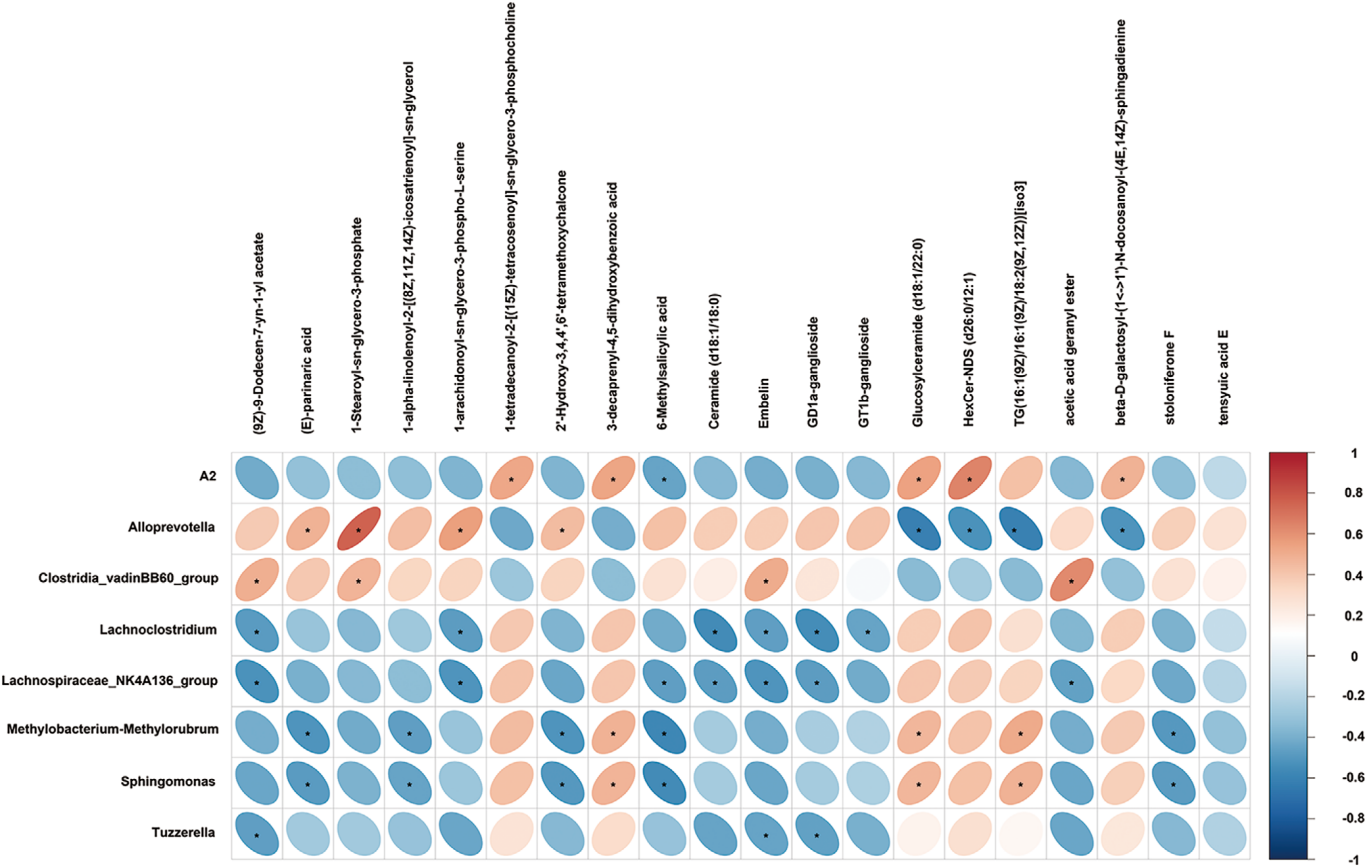
The correlation analysis between gut microbiota and lipid metabolites in hippocampus. The correlation coefficient between gut microbiota at the genus level and hippocampal lipid metabolite levels was indicated by the heatmap. The positive correlation was marked with red, while the blue represents the negative correlation. Pearson statistical method, *p < 0.05.

Pearson correlation was used to associate the diverse taxonomic groups at the genus level with hippocampal lipids (Figure 11). *Alloprevotella* was positively correlated with (E)‐parinaric acid, 1‐stearoyl‐sn‐glycero‐3‐phosphate, 1‐arachidonoyl‐sn‐glycero‐3‐phospho‐L‐serine, 2′‐hydroxy‐3,4,4′,6′‐tetrameth oxychalcone, while *Alloprevotella* was negatively correlated with glucosylceramide (d18:1/22:0), HexCer‐NDS (d26:0/12:1), TG(16:1(9Z)/16:1(9Z)/18:2(9Z,12Z)) [iso3],beta‐D‐galactosyl‐(1↔1′)‐N‐docosanoyl‐(4E,14Z)‐sphingadienine. *Lachnoclostridium* had a negative correlation with 1‐arachidonoyl‐sn‐glycero‐3‐phospho‐L‐serine, ceramide (d18:1/18:0), embelin, GD1a‐ganglioside, and GT1b‐ganglioside. Moreover, *Lachnospiraceae_NK4A136_group* was negatively correlated with (9Z)‐9‐Dodecen‐7‐yn‐1‐yl acetate, 1‐arachidonoyl‐sn‐glycero‐3‐phospho‐L‐serine, 6‐methylsalicylic acid, ceramide (d18:1/18:0), embelin, GD1a‐ganglioside, acetic acid geranyl ester. *Tuzzerella* was negatively correlated with (9Z)‐9‐dodecen‐7‐yn‐1‐yl acetate, embelin, and GD1a‐ganglioside. In addition, we also observed that *Methylobacterium–Methylorubrum* was positively correlated with 3‐decaprenyl‐4,5‐dihydroxybenzoic acid, glucosylceramide (d18:1/22:0), TG(16:1(9Z)/16:1(9Z)/18:2(9Z,12Z))[iso3] and was negatively correlated with(E)‐parinaric acid, 1‐alpha‐linolenoyl‐2‐[(8Z,11Z,14Z)‐icosatrienoyl]‐sn‐glycerol, 2′‐Hydroxy‐3,4,4′,6′‐tetramethoxychalcone, 6‐methylsalicylic acid, and stoloniferone F.

## 4 Discussion

BBR, a bioactive ingredient extracted from barber plants, was previously reported to restore the expression of c‐fos expression and neuronal discharge during PTZ‐induced epileptic seizures [28]. However, the potential mechanism of it is unclear. Our present work discloses the critical role of dysregulation of the gut microbiome in epilepsy and, more importantly, draws a conclusion that treatment with BBR results in the satisfactory amelioration of epileptic seizures via reshaping gut microbiota and associated lipid compositions in the colon and hippocampus. In addition, the protective effect of BBR against KA‐induced epileptic seizures is observed at different time points.

The role of gut microbiota in epilepsy has attracted increasing attention recently. Substantial evidences from animal studies and clinical analysis support that dysbiosis in the gut is associated with epilepsy [30, 31]. Meanwhile, in Mendelian randomization analysis, it was also pointed out that gut microbiota was closely related to epilepsy [32]. In the pilocarpine‐induced epilepsy mouse model, 16sRNA sequencing combined with nuclear magnetic resonance metabolite analysis showed that different gut microbes were associated with some metabolites that maintain epileptic brain activity [33]. Recently, in a deeper intestinal microscopic mechanism, it was found that gut microbiota metabolites mediate the Bax gene to reduce neuronal apoptosis through the cGAS/STING axis in epilepsy [34]. In short, these data all confirm that gut microbiota may be involved in the pathophysiological process of epilepsy.

Fecal microbiota transplantation (FMT) is the most effective strategy for reconstructing the gut microbiota and is considered to treat *Clostridium difficile* infection [35], inflammatory bowel disease [36, 37, 38], constipation, and other diseases [39]. He et al. reported the first case of epilepsy symptoms in Crohn's disease (CD) patients treated with FMT. Intervention with FMT alone for more than 20 months is previously reported to completely block seizures [40]. In our present study, after clearance of bacteria with antibiotics, transplantation with the gut microbiota of NC or BBR treatment in mice is found to significantly attenuate seizure activity including seizure score, number of seizures, and seizure duration. It indicates that the BBR‐treated microbiota provides seizure protection in mice. Olson et al. [33] observed that a germ‐free mouse model of temporal lobe epilepsy is resistant to ketogenic diet treatment and the seizure threshold is elevated in pathogen‐free mice after transplantation with ketogenic diet‐treated bacteria or long‐term administration of *Akkermansia muciniphila* , *Parabacteroides merdae* , and *P. disasonis*. Significantly reduced survival neurons in the hippocampal CA1 and CA3 subregions were also observed in the hippocampal tissue of KA‐treated mice, suggesting that the successful establishment of acute seizure mouse model in this study. BBR significantly protects neurons from damage. However, the clearance of gut microbiota significantly reduced the neuroprotective effect of BBR (Figure 2D).

Gut microbiota dysbiosis has been recognized as one of the important pathogenic factors for epilepsy. A study has compared the gut microbiota of children with refractory epilepsy and healthy controls, especially in terms of microbial community richness index and relative abundance of specific bacterial taxa [24]. It was found that the number of *Bacteroidetes* in the epilepsy group was lower than that in the healthy group, while the number of *Actinobacteria* was higher than that in the healthy group. The epileptic subjects had microbiota richness index 1.6 to 1.7‐fold lower than the healthy controls and harbored a unique species composition [24]. Lindefeldt et al. [41] found that the abundance of *Bacteroidetes* and *Proteobacteria* phyla increased in epilepsy patients, while the abundance of *Firmicutes*, *Cerrucomicrobiota*, and *Furvacheota* phyla decreased.

*Firmicutes* and *Bacteroidetes* are the main communities in the intestine, accounting for 90% of the gut microbiota [42]. The F/B ratio is widely believed to have a significant impact on maintaining normal intestinal homeostasis [43]. BBR increased the abundance of *Bacteroidota* and decreased the abundance of *Firmicutes* and *Actinobacteriota* in the colon contents of mice. *Bacteroid* species, including *Bacteroides fragilis* , produce gamma‐aminobutyric acid (GABA), which is a major inhibitory neurotransmitter [44]. *Bacteroidota* plays a role in seizure regulation, regulating the secretion of IL‐6 and IL‐17 in dendritic cells, a process associated with the severity of seizure, and producing SCFAs [45]. *Bacteroidota* mainly produced acetate and propionate [46], which were the main short‐chain fatty acids (SCFAs) in the colon [47]. SCFAs induce the production of IL‐22 by innate lymphoid cells (ILCs) and CD4 T cells, which is essential for the maintenance of intestinal mucosal immunity [48]. Furthermore, acetate can mediate anti‐inflammatory effects through the GPR43 signaling pathway [46]. SCFAs, including butyrate esters produced by intestinal bacteria, have local and systemic anti‐inflammatory effects, and reducing the production of SCFAs by microorganisms due to dysregulation contributes to various pathological conditions [49]. Safak and colleagues also found that adult patients with focal epilepsy had a reduced number of groups that produced butyrate [50]. Therefore, it is speculated that BBR‐regulated seizures by increasing the abundance of *Bacteroidota* to promote the production of GABA and SCFAs in the colon.

In drug‐resistant epilepsy (DRE), a higher microbial alpha diversity and a significant increase in *Actinobacteria* at the phylum level and *Enterococcus*, *Anaerostipes*, *Bifidobacterium*, *Bacteroides*, and *Blautia* at the genus level were observed [51]. In the epilepsy‐enriched genera, *Bifidobacterium*, *Akkermansia*, *Actinomyces*, and *Enterococcaceae* decreased after KD treatment. At the genus level, BBR resulted in an increase in *Prevotellacea_NK3B31_group* (Figure 3A,B) and a decrease in the abundance of *Lachnospiraceae_NK4A136_group*, *Bifidobacterium*, and *Lactobacillus*. It indicates that BBR causes the reduction of the epilepsy‐enriched genera, including *Actinobacteriota* and *Bifidobacterium*.

Gong et al. observed higher microbial alpha diversity and a significant increase in *Actinobacteria* at the phylum level and *Enterococcus*, *Anaerostipes*, *Bifidobacterium*, *Bacteroides*, and *Blautia* at the genus level in the children with DRE. After six months of KD treatment, the abundance of eight epileptic‐associated genera was reversed, with a decrease in *Bifidobacterium*, *Akkermansia*, *Enterococcaceae*, and *Actinomyces* and increases in *Subdoligranulum*, *Dialister*, and *Alloprevotella*. The genera *Sub‐doligranulum*, *Dialister*, and *Alloprevotella* might be considered as potential taxa for the KD's antiepileptic effects and epilepsy protection [51]. At the genus level, we further found that BBR increased the abundance of *Alloprevotella* at the genus level and decreased the abundance of *Lachnoclostridium*, *Lachnospiraceae_NK4A136_group*, *Tuzzerella*, and *Methylobacterium_Methylorubrum* at the genus level. We further confirmed the increased abundance of *Alloprevotella* through qPCR. *Alloprevotella* is another genus of the *Prevotellaceae*, belonging to Gram‐negative, obligate anaerobic bacilli. It had the ability to ferment carbohydrates and produce SCFAs (acetate and butyrate) [52]. Moreover, *Alloprevotella* had anti‐inflammatory effects [53, 54]. In summary, BBR may attenuate epileptic seizures by increasing the abundance of *Alloprevotella*.

Metabolites from gut microbiota are key molecular mediators between the microbiota and the host. Wang et al. [55] found BBR may supply H through dihydroberberine (reduced BBR produced by bacterial nitroreductase) and promote BH4 production from dihydrobiopterin; the increased BH4 enhances TH activity, thereby accelerating L‐dopa production by gut bacteria. Previous studies have found that the colonization of ASD microbiota is sufficient to induce hallmark autistic behavior. Moreover, treatment of ASD mouse models with candidate microbial metabolites can improve behavioral abnormalities and regulate the excitability of brain neurons [56]. Zhu et al. found that BBR showed significant therapeutic effects in the PTZ‐induced chronic epilepsy model after long‐term treatment. Through the joint analysis of network pharmacology and lipidomics, it was also found that high‐potential metabolites between the BBR group and the model group including L‐tryptophan, 5‐hydroxyindoleacetic acid, guanidoacetic acid, hippuric acid, L‐phenylalanine, L‐tyrosine, and glycine. Furthermore, phenylalanine metabolism and tryptophan metabolism may be powerful pathways for BBR to treat epilepsy [57].

Through lipidomics analysis, we identified 338 significantly different lipids in the colon contents of NC and BBR treatments, of which 101 were upregulated and 237 were downregulated. Moreover, 8 biomarkers affected by BBR were screened. KEGG pathway analysis revealed that glycine, serine and threonine metabolism, and glycerophospholipid metabolism were the potential pathways. 160 significantly different lipids were identified in the hippocampus of NC and BBR, of which 131 were upregulated and 29 were downregulated. In addition, 13 biomarkers of BBR regulation were screened. KEGG pathway analysis revealed that arachidonic acid metabolism, serotonergic synapse, and ferroptosis were the potential pathways in hippocampal tissue samples. Furthermore, we conducted a correlation analysis between lipidomics and gut microbiota and found that *Alloprevotella* reduced the levels of (23S)‐methyl‐3alpha,7alpha,12alpha‐trihydroxy‐5beta‐cholan‐24‐oic acid, (3beta,24R)‐Ergost‐5‐en‐3‐yl (13Z,16Z)‐13,16‐docosadienoate, MFCD00678983, N‐icosanoylsphingosine 1‐phosphate in the colon (Figure 10). *Allophorotella* increased the content of (E)‐parinaric acid, 1‐Stearoyl‐sn‐glycero‐3‐phosphate, 1‐arachidonoyl‐sn‐glycero‐3‐phospho‐L‐serine, 2′‐Hydroxy‐3,4,4′,6′‐tetramethoxychalcone and reduced the content of glucosylceramide (d18:1/22:0), HexCer‐NDS (d26:0/12:1), TG(16:1(9Z)/16:1(9Z)/18:2(9Z,12Z))[iso3], beta‐D‐galactosyl‐(1↔1′)‐N‐ docosanoyl‐(4E,14Z)‐sphingadienine in the hippocampus (Figure 11). Further research is indispensable to investigate the relationship between these screened differential metabolites and epilepsy.

## 5 Conclusion

Our present work indicates that BBR alleviates acute epileptic seizures induced by KA in mice by increasing the abundance of *Bacteroidetes* and *Alloprevotella*, regulating the concentration of SCFAs. In addition, 21 biomarkers are also identified in the colon and hippocampus following BBR treatment. These findings suggest that BBR exerts promising protection against KA‐induced acute epileptic seizures in mice through remodeling gut microbiota to regulate lipid metabolism in the colon and hippocampus. BBR shows great promise for the treatment of acute epileptic seizures and drug‐resistant epilepsy.

## Author Contributions

**Wen‐Ting Dai:** data curation, investigation, methodology, visualization, writing – original draft. **Yong Zhu** and **Zui‐Ming Jiang:** data curation, investigation, methodology. **Zhao‐Qian Liu**, **Xiao‐Yuan Mao** and **Yi Xiang:** funding acquisition, methodology, project administration, resources, supervision, writing – review and editing.

## Conflicts of Interest

The authors declare no conflicts of interest.

## Data Availability

The data that support the findings of this study are available on request from the corresponding author.
